# Supplementary material for: Risk factors and barriers to case management of neonatal pneumonia: protocol for a pan-India qualitative study of stakeholder perceptions
Source: BMJ Open. 2017 Sep 29;7(9):e017403. doi: 10.1136/bmjopen-2017-017403 (PMC5640148; doi:10.1136/bmjopen-2017-017403)
Supplement: Author's manuscript [file bmjopen-2017-017403.draft_revisions.pdf]

BMJ Open

BMJ Open is committed to open peer review. As part of this commitment we make the peer review history of every article we publish publicly available.

When an article is published we post the peer reviewers' comments and the authors' responses online. We also post the versions of the paper that were used during peer review. These are the versions that the peer review comments apply to.

The versions of the paper that follow are the versions that were submitted during the peer review process. They are not the versions of record or the final published versions. They should not be cited or distributed as the published version of this manuscript.

BMJ Open is an open access journal and the full, final, typeset and author-corrected version of record of the manuscript is available on our site with no access controls, subscription charges or pay-per-view fees (http://bmjopen.bmj.com).

If you have any questions on BMJ Open's open peer review process please email info.bmjopen@bmj.com

BMJ Open

Risk factors and barriers to case management of neonatal pneumonia: a pan-India qualitative study of stakeholder perceptions (Protocol)

Journal:	*BMJ Open*
Manuscript ID	bmjopen-2017-017403
Article Type:	Protocol
Date Submitted by the Author:	25-Apr-2017
Complete List of Authors:	Sreekumaran Nair, N; Manipal University, Department of Statistics and Public Health Evidence South Asia (PHESA)<br>Chronos, Support; Bill & Melinda Gates Foundation,<br>Lewis, Leslie; Manipal University Kasturba Medical College Manipal<br>Godinho, Myron ; Manipal University, Public Health Evidence South Asia (PHESA)<br>Murthy, Shruti; Manipal University, Public Health Evidence South Asia (PHESA)<br>Lakiang, Theophilus; Manipal University<br>Venkatesh , Bhumika; Manipal University
**Primary Subject Heading**:	Public health
Secondary Subject Heading:	Paediatrics
Keywords:	Infant, Pneumonia, Qualitative study, Protocol, Barriers, Management

SCHOLARONE™ Manuscripts

Risk factors and barriers to case management of neonatal pneumonia: a pan-India qualitative study of stakeholder perceptions (Protocol)

N. Sreekumaran Nair[1], Leslie Edward Lewis[2], Theophilus Lakiang[3*], Myron Godinho[3**], Shruti Murthy[3***], Bhumika T. Venkatesh[4]

[1] Director, Public Health Evidence South Asia (PHESA), & Professor of Biostatistics and Head, Department of Statistics, Manipal University, Manipal. nsknairmanipal@gmail.com

[2] Professor and Head, Neonatology Unit, Department of Pediatrics, Kasturba Medical College, Manipal University, Manipal. leslie.lewis@manipal.edu

[3] Research Associate, Public Health Evidence South Asia (PHESA), Manipal University, Manipal;

*theo.lakiang@gmail.com ;

**myrongodinho@gmail.com;

*** itzshruti@gmail.com;

[4]Assistant Professor, Public Health Evidence South Asia (PHESA), Manipal University, Manipal; meetbhumika123@gmail.com

Correspondence to:
Dr. N. Sreekumaran Nair
Director, Public Health Evidence South Asia (PHESA), Ground Floor, Manipal EDU Building, and Professor of Biostatistics and Head, Department of Statistics,
Level 6, Health Science Library Building,
Manipal University, Manipal-576104, Karnataka, India
Email address: nsknairmanipal@gmail.com
Telephone number: +91-8202922407

For peer review only

Risk factors and barriers to case management of neonatal pneumonia: a pan-India qualitative study of stakeholder perceptions (Protocol)

Abstract

Introduction: Over half of India's under-five deaths occur among neonates, and pneumonia is one of the deadliest neonatal infectious disease. Despite simple, inexpensive case management strategies being available, pneumonia continues to cause significant mortality and morbidity among neonates. Understanding the perceptions and experiences of stakeholders of neonatal care can help find solutions to barriers to care and design tailored strategies for controlling neonatal pneumonia.

Methods and analysis: This multi-regional qualitative study is the first of its kind, and is part of a mixed methods investigation of neonatal pneumonia in India. Eligible participants include health care providers, programme officers, academicians, representatives of NGOs/ bilateral agencies and policy makers. They will be recruited purposively from rural and urban, public and private, and facility and community health care settings across six Indian regions. Within each region, a minimum of one state will be selected. Districts will be based on neonatal mortality indicators. District selection will also be subject to feasibility at the time of conducting the study. We plan to conduct in-depth interviews (IDI) and Focus Group Discussions (FGD) with participants. IDI and FGD will focus on the following domains: (a) burden, (b) risk factors, (c) case management, (d) treatment guidelines, (e) barriers to case management, (f) recommendations. The number of interviews will depend on the information saturation. For face-to-face interviews, an interview and a note taker will be present at each interview. Interviews will be audio- recorded with prior written consent and transcribed verbatim. Principles of thematic analysis will be applied for qualitative data analysis on RQDA.

Ethics and dissemination: The study will be conducted according to the principles in the Declaration of Helsinki. The protocol has been approved by the Health Ministry Screening Committee (HMSC), Government of India and the Institutional Ethics Committee at the host institution [DCG(I) Registration number: ECR/191/Inst/KL/2013]. Confidentiality and privacy of the participant will be maintained at every stage of the research by assigning unique identification numbers, anonymizing records, delinking all files with potential identifiers and data cleaning procedures. The findings of the study will be shared with all stakeholders of

For peer review only

this research including the participants. Knowledge dissemination workshops will be conducted to ultimately transfer the evidence tailored to the stakeholder (e.g. policy briefs, publications, information booklets, etc).

Keywords: Neonate, Pneumonia, Qualitative study, Protocol, Barriers, Management

Strengths and limitations of the study

- A pan-India study covering all regions of India and wide range of stakeholders of the public and private, rural and urban neonatal health system
- Contribution of knowledge about barriers to management and control of pneumonia among neonates in the Indian context
- Final selection of states and districts from each region will depend on the feasibility of access and willingness of participants in the district.

Introduction

In recent decades in India, the neonatal mortality rate has declined more slowly than the post-neonatal mortality rate, and now over half of India's under-five deaths occur in the neonatal age group. 17% of these are attributed to pneumonia, making it the most deadly neonatal infection.[1] In the international effort to curb child deaths, addressing neonatal pneumonia in India is clearly an issue of global concern.

Developing countries contribute to around 151 of the annual 156 million under-five pneumonia cases. India is the largest contributor to global pneumonia deaths, accounting for around 43 million cases per year. Neonatal pneumonia differs from childhood pneumonia in many ways, and is classified as 'early onset' or 'late onset', corresponding to the two major environments from which the infectious agent may be contracted: the intrauterine environment and birth canal, and the external environment. [2] Moreover, differences in infective agents result in a number of characteristic variants of neonatal pneumonia.[3]

The likelihood of occurrence can be augmented by risk factors and determinants. In India, economic compromise, poor immunization status, malnutrition and indoor air pollution are

For peer review only - http://bmjopen.bmj.com/site/about/guidelines.xhtml

some of the major risk factors for developing pneumonia.[4] Studies have previously reported that low birth weight, inadequate antenatal care, home birthing, untrained personnel, and other factors are associated with neonatal pneumonia.[5]

Effective case management is a cornerstone of pneumonia control in children. However, studies have reported barriers (e.g. cost and complexities in treatment) to its implementation, especially in low-and-middle income (LMICs) like India, where the burden of pneumonia morbidity and mortality is high.[6] This is compounded by numerous supply (e.g. health system, staff absenteeism, lack of infrastructure) and demand (e.g. local social fact cultural barriers, health care perceptions and experiences) issues, which plague public health systems in developing countries.[7 8]

Understanding context-specific factors and barriers for management and control of neonatal pneumonia is essential to design effective strategies for Each stakeholder at different levels of healthcare system (policymakers, healthcare providers, grass root level workers, informal care givers) experiences a different functional position in the healthcare delivery system, and is therefore able to offer in-depth, diverse and comprehensive perspectives of neonatal care, all of which are essential to attain a more complete understanding of neonatal pneumonia. The holistic understanding may help address the neonatal health by involving multiple actors and thus multiple factors to address neonatal health, a 'complex' problem. [9] Qualitative research has successfully been used to provide evidence to inform government-stated policy priorities in developing countries, by promoting an understanding of local problems and solutions to develop effective and sustainable interventions.[8]

Qualitative studies have previously focused on perceptions of caregivers on barriers and factors related to pneumonia in childhood. However, empirical evidence largely exists for the childhood pneumonia and neonatal sepsis. There is a need for a study which captures the variety and depth of context-specific perceptions and experiences associated with neonatal pneumonia in India's various regions.

Aims: This qualitative study is a part of a larger mixed-methods research project which also involves three systematic reviews on the same domains of inquiry as this study. This study will provide an in-depth and comprehensive understanding of neonatal pneumonia from

stakeholders at different health system levels and settings across India. The findings of this study are planned to be synthesized with the evidence from the three reviews. The synthesized findings are anticipated to fill the evidence gap related to an understanding of the occurrence and case management of neonatal pneumonia in the Indian context. In addition, the recommendations provided by the stakeholders from their experience will serve as essential inputs to inform policy and design strategies to address neonatal health disparities more comprehensively across the diverse range of contexts present in this country.

Objectives:

1. To identify the risk factors of pneumonia in the neonatal age group (0-28 days) in the Indian context.
2. To understand pertinent barriers to the case management of neonatal pneumonia in Indian health care facilities.
3. To understand factors that could facilitate the control of neonatal pneumonia in India

Methods and analysis:

Study Design: A qualitative study will be conducted to explore and understand the perceptions and experiences of various stakeholders regarding the following domains of interest:

- risk factors associated with neonatal pneumonia in the Indian context;
- barriers to the case management of neonatal pneumonia;
- recommendations for control and management of neonatal pneumonia in India

Study Setting: India, the largest country in South Asia, is the world's second most populous country, with about 1.03 billion people in 29 States and 7 Union Territories.[10 11] Eleven percent of the total population is under 4 years of age.[1] Over two-thirds of the population live in rural areas which continue to lag far behind urban India in socioeconomic progress.[12] This disparity is reflected in the neonatal health statistics: while Uttar Pradesh has a neonatal mortality rate (NMR) of 49 deaths per thousand live births (DPTLB) which is higher than the regional aggregate for sub-Saharan Africa, Kerala boasts a single digit NMR of 7 DPTLB, resembling a considerably more developed nation.[13] Even within a state, statistics

For peer review only

vary greatly between districts;[14] with Shrawasti and Hamirpur illustrating the contrast in Uttar Pradesh, with NMRs of 50.8 and 17.6 DPTLB, respectively.[15]

This study will be conducted during 2016-17. We will recruit participants from multiple regions within India, representing both urban and rural, as well as facility-based and community-based neonatal care. To achieve this, we adapted a classification proposed in a previous study on neonatal mortality in India, where states were grouped into six regions: North, South, Central, West, East and Northeast.[16]. Within each region, a minimum of one state will be selected. Using the most recent neonatal mortality estimates available,[15] we generated a series of district-level maps of neonatal mortality indicators (published elsewhere). Within the selected state, districts were grouped by NMR from published secondary data. We identified a total of four districts in each state: two with the lowest NMR (best performers), and two with the highest NMR (worst performers), (figure 1). We plan to recruit participants from one of each of the best performing and worst performing districts respectively. All geographical areas suspected to be conflict-prone, unsafe or inaccessible will be identified and excluded. District selection will also be subject to feasibility (e.g. after contacting key informants and liaisons in identified districts and discussing with advisory committee members). From these district(s), participants from urban and rural, public and private facility and community health care settings at different levels of the health care system will be recruited. This strategy was developed to recruit participants from contrasting settings, allowing for a wider range of perspectives, in order to build a comprehensive picture of neonatal pneumonia in India.

For peer review only

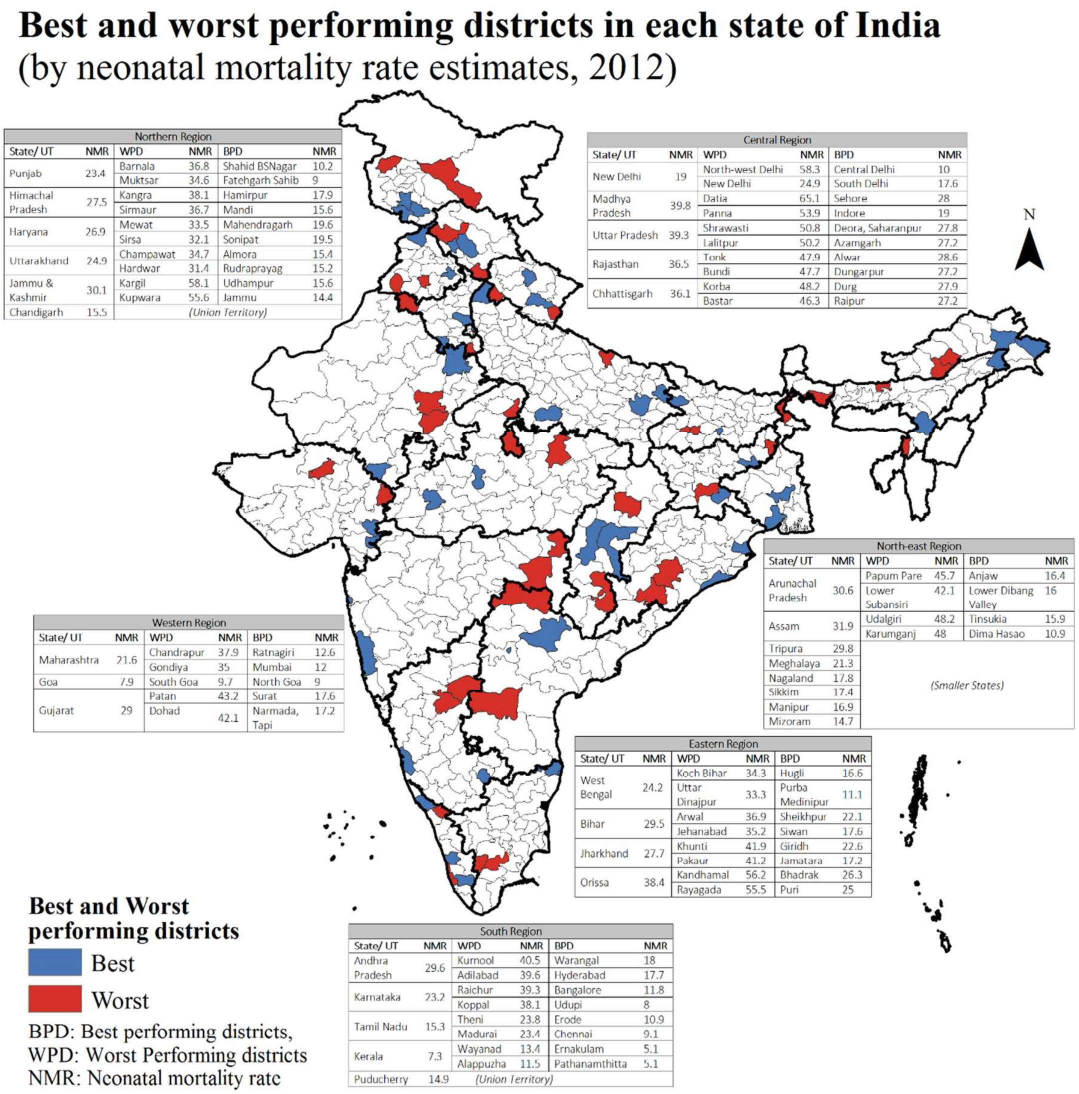

Figure 1. A map of India depicting the two best and worst performing districts, by NMR, in each Indian state.*

** adapted, with permission, from data published by Ram U, Jha P, Ram F, et al. Neonatal, 1–59 month, and under-5 mortality in 597 Indian districts, 2001 to 2012: estimates from national demographic and mortality surveys. The Lancet Global Health 2013;1(4):e219-e26.*

Participants

Through discussions with advisory members, it was agreed that as far as possible, we would target stakeholders from every component and level of the health system. We aim to include the following stakeholders in our study:

1. Programme officers (e.g. RMNCH+A/ IMNCI, State/District Health Officers)

2. Neonatologists, Pediatricians, Medical officers, Neonatal nurses, GNMs (General Nurse Midwives), ANMs (Auxiliary nurse midwives) and other facility-based staff.
   It is anticipated that much information can be obtained on risk factors for neonatal pneumonia (hospital-acquired and ventilator-associated) and barriers to facility-based case management of neonatal pneumonia.
3. Community health workers (e.g. Accredited Social Health Activists [ASHA] and state-specific equivalents), AnganWadi Workers (AWW).
   It is anticipated that community-based staff will provide valuable insights into the burden, risk factors and barriers to the case management of neonatal pneumonia, as well as other related health services and schemes available for neonatal care.
4. NGO/ bilateral agencies (e.g. UNICEF) representatives.
5. Academicians (e.g. Indian Academy of Pediatrics' members)
6. Policymakers (e.g. Ministry of Health and Family Welfare representatives)

Recruitment: A purposive sampling approach will be employed to include participants from different settings and at various levels of the health system. Health care providers from private and public setups, at the community and facility at the primary, secondary and tertiary health care level will be included. Participants at different health system levels i.e. responsible for policy making and implementation will also be identified and recruited.

To ensure successful participation, participants will be identified by liaising with governmental, non-governmental, bilateral and academic organizations who work in maternal and child health fields in the selected regions. Contact networks of the project and advisory members will also be utilized to facilitate this process. The participants will then be invited through personal telephonic calls and a formal invitation letter which will include the project brief. On consenting to participate in the research, the time, location and mode (face-to-face/ tele-interviews) of IDI will be ascertained through consultation with the participants. All efforts will be made to interview the participant in their natural setting. A minimum of 24 neonatologists/ pediatricians, 12 neonatal nurses/ ANMs, 12 medical officers and 6 policy makers will be interviewed in total. However, the final numbers will be determined by attaining information saturation.

Data collection methods: In-depth interviews (IDI) and focus group discussions (FGD) will be used to collect data. While IDI will comprise the main mode of data collection, we anticipate

For peer review only

that focus group discussions (FGDs) might become necessary in some instances where nurses or community health workers may be involved as participants. When a minimum of four participants of the same category (e.g. nurses or ASHAs) are available and in depth interviews may not seem feasible (e.g. time constraints per participant), a FGD will be conducted. Similar guides will be used for IDI and FGD.

A detailed IDI/FGD guide will be formulated after literature review, in consultation with clinical experts and qualitative research specialists. The guide will be customized to the level of the stakeholder to allow questioning strategy to be more flexible. The IDI/FGD guide which will contain the lists of issues we want to explore and will include open ended questions on the following themes relating to neonatal pneumonia in India (a) burden, (b) risk factors, (c) case management, (d) treatment guidelines, (e) barriers to case management, (f) recommendations. The guide for the community health workers will focus on community-acquired pneumonia in neonates and additionally include other relevant health schemes/ services related to community-acquired neonatal pneumonia in India. Supporting documents will include development of (i) survey form which collects data on participant details (socio-demographic information and duration of experience), consent details and interview details, and (ii) observations and field notes summary form. The guide will be pilot-tested before we proceed with the data collection in order to ensure that the guide is vivid and participants understanding of the concept and language. Any modification required will be taken into consideration after pilot-testing of the guide to be able to generate more in-depth information.

Two adequately qualified and trained research associates will be present during the interview; the primary researcher will lead the interview, and a second researcher will be responsible for recording the interview, making observations and taking field notes. Interviews will be conducted either through face to face interaction or through online sources like Skype, teleconference, etc. (based on the convenience of the interviewee).

Recording: Interviews will be conducted in a language which the participant is comfortable speaking. Each interview is anticipated to last for 60 to 90 minutes. The number of interviews per participant will depend on information saturation. For face-to-face interviews, an interview and a note taker will be present at each interview. For tele-

For peer review only

interviews, only the interviewer will be present at each interview. Interviews will be recorded on a portable, electronic voice recorder (Sony ICD-PX440), and transferred to a computer for storage and transcription. Field notes will be maintained for every interview which will highlight the reflections on the interview process, non-verbal cues of the interviewee as well as the initial themes that emerge as a result of the interview.

Data management

Data organization: A unique identification code will be assigned to audio files. Audio recording of each interview will be assigned with a similar unique identification code to the participant's identification code. Naming of the file will help the team to easily locate and retrieve the file whenever required for transcription. All transcripts will be named after participation ID and date and place of interview. Then the transcripts of each interview will be saved along with the informed consent of that particular participant.

Data transcription: All interviews will be recorded (with prior consent) and transcribed verbatim (involves active and repeated listening and observation of the data collected). Interviews will first be transcribed in the language of the interview and then translated into English. The transcripts will be supplemented with field notes, if and when necessary. The English transcripts for interviews other than those in English will be back translated into that language at a later stage for quality assurance purposes. To ensure quality, 10% of the transcripts will be checked with the tape-recorded interviews by a researcher other than the interviewer.

Data storage: A master archival log and an archival information sheet will be developed beforehand, all audio files and transcripts lists will be entered into the archival sheet. The archival sheet such will contain details such as date, location, interviewer's name, name of the person who transcribed the data etc. Backup of all transcript files to a secured external device which can be accessible only to the research team

Data protection: Audio files, transcripts, etc. will be shared only amongst the research team and as required by regulatory laws of the Government of India. The files will be password protected and can only be accessible by agreed members of the research team. Computer

For peer review only

files need to be kept securely and can only be shared based on the terms and condition that the participant agreed upon in the informed consent.

Data ownership: Research team, funding agency and stakeholders will retain ownership of the data. Further clarification on data ownership will be discussed.

Data Analysis: The transcript will be cleaned only when it has to do with the identity of the participants. Where possible, the data from the transcripts will be organized in the form of a chart or table that will data allows us to easily analyze the response to each question individually, in order to make it easier to pick out concepts and themes. Thematic analysis of the transcripts will be done. Codes will be developed by reading and re-reading the transcripts. For this, specific words/phrases/ideas/opinion/issues related to the objective of the study that occurs repeatedly in the data will be identified. The interviewers will make note of the different ideas as the different responses are read through. Different codes will be explored and listed till information is saturated. A code book will be developed to list and define the codes identified based on the objective of the study. Two of the research team members and an independent qualitative research advisor of the project will check the two cycles of coding using the IDI guide as reference. The coders will then meet to discuss the coding schemes and resolve differences. Re-labeling of the codes will be done if and where necessary. Next, similar codes will be grouped and categorized in a meaningful way. This will subsequently lead to generation of important categories and concepts related to the main themes of the study. Each response may be associated to one or more theme. Hence, different categories can be classified under one main over-arching theme. In addition to already identified themes, themes will be derived, if any, from the categories. The data will be managed by the use of R QDA package. This software will facilitate the organization and management of datasets at every stage of qualitative data analysis.

For peer review only

Ethics and dissemination

Ethical Approval: The study will be conducted according to the principles in the Declaration of Helsinki. The protocol has been approved by the Health Ministry Screening Committee (HMSC), Government of India and the Institutional Ethics Committee at the host institution [DCG(I) Registration number: ECR/191/Inst/KL/2013].

Informed Consent: Participation will be entirely voluntary. Informed consent will be mandatory for participation in the study. For face-to-face interviews, written informed consent will be obtained. For tele-interviews, verbal informed consent will be obtained. Where recording is denied, hand written notes will be taken by the researchers. [17]

Confidentiality: Confidentiality and privacy of the participant will be maintained at every stage of the research. A unique identification code will be given to each participant. Recordings of the interviews will be assigned only the number and cannot be linked to the participant's identifiers. During data cleaning, all potential identifiers will be removed to create a 'clean' data set.[18] The data will be kept anonymous at all stages of the study. Participants will remain unidentifiable in all transcripts, and in all reports from this study.

Dissemination: The findings of the study will be shared with all stakeholders of this research including the participants. Knowledge dissemination workshops will be conducted with relevant stakeholders to ultimately transfer the evidence tailored to the stakeholder (e.g. policy briefs, publications, information booklets, etc).

Quality Control and Reporting

Reporting of the qualitative study will be informed by two guidelines: The Standards for Reporting Qualitative Research (SRQR)[19] and the Consolidated criteria for reporting qualitative research (COREQ).[20]

Acknowledgements: We would like to thank the following individuals for their continuous support and guidance during this process of protocol development: Dr. Ajay Bailey, Assistant Professor, Faculty of Spatial Sciences, University of Groningen, The Netherlands; Dr. Shrinivas Darak, Senior researcher, PRAYAS, Pune, Maharashtra; Dr. Manoj Das, Director Projects, The INCLEN Trust International, New Delhi; Dr. B. Shantharam Baliga, Professor, Department of Paediatrics, Kasturba medical college, Mangalore, Karnataka; Dr. K.K. Diwakar, Professor and Head, Department of Neonatology, Associate Dean, Malankara Orthodox Syrian Church Medical College, Kerala and Dr. Unnikrishnan B., Associate Dean and Professor, Department of Community Medicine, Kasturba Medical College, Mangalore. We also thank Public Health Evidence South Asia (PHESA) and Manipal University, Manipal for providing the necessary institutional and infrastructural support for the project. We

For peer review only

would like to thank The INCLEN Trust International, New Delhi, and The Bill and Melinda Gates Foundation for the financial support which made this project possible.

Contributorship statement: SN, BV and LL conceived the research idea and reviewed the manuscript. SN and LL provided overall technical guidance. In addition, LL assisted in developing the in-depth interview (IDI) guides. TL, MG and SM designed the protocol, drafted the manuscript, and developed and pilot-tested the IDI guides. All authors approved the final version of the manuscript.

Funding: This project is supported by a grant from Bill and Melinda Gates Foundation (grant OPP1084307) to The INCLEN Trust International and sub-grant to Manipal University (subgrant INC2015GNT004). However, the views expressed through this project do not necessarily represent the views of the Bill and Melinda Gates Foundation, The INCLEN Trust International, or Manipal University.

Competing Interests: All authors have completed the ICMJE uniform disclosure form at www.icmje.org/coi_disclosure.pdf and declare: all authors had financial support (grants) from Bill and Melinda Gates Foundation (grant OPP1084307) to The INCLEN Trust International and sub-grant to Manipal University (subgrant INC2015GNT004)., during the conduct of the study and for the submitted work; no financial relationships with any organisations that might have an interest in the submitted work in the previous three years; no other relationships or activities that could appear to have influenced the submitted work.

Data sharing statement: Due to ethical concerns, supporting data (e.g. interview transcripts) cannot be made publicly available. All other data supporting this study (e.g. coding structure) will be provided as final results and as supplementary material accompanying the manuscript of the study's final results

For peer review only

References

1. World Health Organisation (WHO). Neonatal and Child Health Profile, India: Department of Maternal Newborn Child and Adolescent Health 2013 [accessed 2016 19/1/2016]. Available from: http://www.who.int/
2. Dennehy P. Respiratory infections in the newborn. *Clinics in perinatology* 1987;14(3):667-82.
3. Webber S, Wilkinson A, Lindsell D, et al. Neonatal pneumonia. *Archives of disease in childhood* 1990;65(2):207-11.
4. Mathew JL, Patwari AK, Gupta P, et al. Acute respiratory infection and pneumonia in India: a systematic review of literature for advocacy and action: UNICEF-PHFI series on newborn and child health, India. *Indian pediatrics* 2011;48(3):191-218.
5. Choudhury A, Nargis S, Mollah A, et al. Determination of risk factors of neonatal pneumonia. *Mymensingh medical journal: MMJ* 2010;19(3):323-29.
6. UNICEF, World Health Organization. Global action plan for prevention and control of pneumonia (GAPP). 2009
7. Patil AV, Somasundaram K, Goyal R. Current health scenario in rural India. *Australian Journal of Rural Health* 2002;10(2):129-35.
8. Bedford KJA, Sharkey AB. Local barriers and solutions to improve care-seeking for childhood pneumonia, diarrhoea and malaria in Kenya, Nigeria and Niger: a qualitative study. *PloS one* 2014;9(6):e100038.
9. Rwashana AS, Nakubulwa S, Nakakeeto-Kijjambu M, et al. Advancing the application of systems thinking in health: understanding the dynamics of neonatal mortality in Uganda. *Health Research Policy and Systems* 2014;12(1):1.
10. The World Bank. World Bank Open Data. Rural Population. 2016 [accessed 2016 15/4/2016]. Available from: http://data.worldbank.org/.
11. Office of the Registrar General & Census Commissioner MoHA, Government of India. . Age structure and Marital Status, Census 2011. Available from: http://censusindia.gov.in/.
12. Mukunthan A. Rural India is far behind Urban India in every Indicator of Progress. Factly.[newspaper article on the Internet]. 2015. [accessed 2016 16/4/2016]. Available from: https://factly.in/rural-india-behind-urban-india-in-progress-indicators/
13. Ministry of Health and Family Welfare, Department of Health and Family Welfare, Governmnet of India.Annual Health Survey. Catalog of Indicators [accessed 2016 1/1/2016]. Available from: https://data.gov.in/catalog/indicators-annual-health-survey.
14. Ministry of Health and Family Welfare, Government of India (GoI). Child Health Division. India Newborn Action Plan. New Delhi; September 2014 [accessed 2016 14/1/2016]. Available from: http://www.newbornwhocc.org/.
15. Ram U, Jha P, Ram F, et al. Neonatal, 1–59 month, and under-5 mortality in 597 Indian districts, 2001 to 2012: estimates from national demographic and mortality surveys. *The Lancet Global Health* 2013;1(4):e219-e26.
16. Million Death Study Collaborators. Causes of neonatal and child mortality in India: a nationally representative mortality survey. *The Lancet* 2010;376(9755):1853-60.
17. World Health Organisation (WHO). Informed Consent Form Templates 2016 [accessed 2016 8/4/2016]. Available from: http://www.who.int/.
18. Kaiser K. Protecting respondent confidentiality in qualitative research. *Qualitative Health Research* 2009;19(11):1632-41.
19. O'Brien BC, Harris IB, Beckman TJ, et al. Standards for reporting qualitative research: a synthesis of recommendations. *Academic Medicine* 2014;89(9):1245-51.
20. Tong A, Sainsbury P, Craig J. Consolidated criteria for reporting qualitative research (COREQ): a 32-item checklist for interviews and focus groups. *International journal for quality in health care* 2007;19(6):349-57.

For peer review only

PRISMA-P (Preferred Reporting Items for Systematic review and Meta-Analysis Protocols) 2015 checklist: recommended items to address in a systematic review protocol*

Section and topic	Item No	Checklist item	Compliance
**ADMINISTRATIVE INFORMATION**			
Title:			
Identification	1a	Identify the report as a protocol of a systematic review	Pg 1& 2
Update	1b	If the protocol is for an update of a previous systematic review, identify as such	Not applicable
Registration	2	If registered, provide the name of the registry (such as PROSPERO) and registration number	Pg 1
Authors:			
Contact	3a	Provide name, institutional affiliation, e-mail address of all protocol authors; provide physical mailing address of corresponding author	Pg 1
Contributions	3b	Describe contributions of protocol authors and identify the guarantor of the review	Pg 10
Amendments	4	If the protocol represents an amendment of a previously completed or published protocol, identify as such and list changes; otherwise, state plan for documenting important protocol amendments	Not applicable
Support:			
Sources	5a	Indicate sources of financial or other support for the review	Pg 10
Sponsor	5b	Provide name for the review funder and/or sponsor	Pg 10
Role of sponsor or funder	5c	Describe roles of funder(s), sponsor(s), and/or institution(s), if any, in developing the protocol	Pg 10-11
**INTRODUCTION**			
Rationale	6	Describe the rationale for the review in the context of what is already known	Pg 4
Objectives	7	Provide an explicit statement of the question(s) the review will address with reference to participants, interventions, comparators, and outcomes (PICO)	Pg 4 Modified accordingly for review of non-interventional studies
**METHODS**			
Eligibility criteria	8	Specify the study characteristics (such as PICO, study design, setting, time frame) and report characteristics (such as years considered, language, publication status) to be used as criteria for eligibility for the review	Pg 4-5 Modified accordingly for

For peer review only

			review of non-interventional studies
Information sources	9	Describe all intended information sources (such as electronic databases, contact with study authors, trial registers or other grey literature sources) with planned dates of coverage	Pg 5-6
Search strategy	10	Present draft of search strategy to be used for at least one electronic database, including planned limits, such that it could be repeated	Pg 6
Study records:			
Data management	11a	Describe the mechanism(s) that will be used to manage records and data throughout the review	Pg 6
Selection process	11b	State the process that will be used for selecting studies (such as two independent reviewers) through each phase of the review (that is, screening, eligibility and inclusion in meta-analysis)	Pg 6-7
Data collection process	11c	Describe planned method of extracting data from reports (such as piloting forms, done independently, in duplicate), any processes for obtaining and confirming data from investigators	Pg 7
Data items	12	List and define all variables for which data will be sought (such as PICO items, funding sources), any pre-planned data assumptions and simplifications	Pg 6-7
Outcomes and prioritization	13	List and define all outcomes for which data will be sought, including prioritization of main and additional outcomes, with rationale	Pg 5
Risk of bias in individual studies	14	Describe anticipated methods for assessing risk of bias of individual studies, including whether this will be done at the outcome or study level, or both; state how this information will be used in data synthesis	Pg 8
Data synthesis	15a	Describe criteria under which study data will be quantitatively synthesised	Pg 8-9
	15b	If data are appropriate for quantitative synthesis, describe planned summary measures, methods of handling data and methods of combining data from studies, including any planned exploration of consistency (such as I^2, Kendall's τ)	Pg 8
	15c	Describe any proposed additional analyses (such as sensitivity or subgroup analyses, meta-regression)	Pg 8-9
	15d	If quantitative synthesis is not appropriate, describe the type of summary planned	Pg 8
Meta-bias(es)	16	Specify any planned assessment of meta-bias(es) (such as publication bias across studies, selective reporting within studies)	Pg 9
Confidence in cumulative evidence	17	Describe how the strength of the body of evidence will be assessed (such as GRADE)	Pg 9

*** It is strongly recommended that this checklist be read in conjunction with the PRISMA-P Explanation and Elaboration (cite when available) for important clarification on the items. Amendments to a review protocol should be tracked and dated. The copyright for PRISMA-P (including checklist) is held by the PRISMA-P Group and is distributed under a Creative Commons Attribution Licence 4.0.**

From: Shamseer L, Moher D, Clarke M, Ghersi D, Liberati A, Petticrew M, Shekelle P, Stewart L, PRISMA-P Group. Preferred reporting items for systematic review and meta-analysis protocols (PRISMA-P) 2015: elaboration and explanation. BMJ. 2015 Jan 2;349(jan02 1):g7647.

For peer review only

BMJ Open

Risk factors and barriers to case management of neonatal pneumonia: Protocol for a pan-India qualitative study of stakeholder perceptions

Journal:	*BMJ Open*
Manuscript ID	bmjopen-2017-017403.R1
Article Type:	Protocol
Date Submitted by the Author:	05-Jul-2017
Complete List of Authors:	Sreekumaran Nair, N; Manipal University, Department of Statistics and Public Health Evidence South Asia (PHESA)<br>Lewis, Leslie; Manipal University Kasturba Medical College Manipal<br>Lakiang, Theophilus; Manipal University<br>Godinho, Myron ; Manipal University, Public Health Evidence South Asia (PHESA)<br>Murthy, Shruti; Manipal University, Public Health Evidence South Asia (PHESA)<br>Venkatesh , Bhumika; Manipal University
**Primary Subject Heading**:	Public health
Secondary Subject Heading:	Paediatrics
Keywords:	Pneumonia, Qualitative study, Protocol, Neonate, Case management, India

SCHOLARONE™
Manuscripts

For peer review only

Risk factors and barriers to case management of neonatal pneumonia: Protocol for a pan-India qualitative study of stakeholder perceptions

N. Sreekumaran Nair[1], Leslie Edward Lewis[2], Theophilus Lakiang[3*], Myron Godinho[3**], Shruti Murthy[3***], Bhumika T. Venkatesh[4]

[1] Director, Public Health Evidence South Asia (PHESA), & Professor of Biostatistics and Head, Department of Statistics, Manipal University, Manipal. sree.nair@manipal.edu, nsknairmanipal@gmail.com

[2] Professor and Head, Neonatology Unit, Department of Pediatrics, Kasturba Medical College, Manipal University, Manipal. leslie.lewis@manipal.edu

[3] Research Associate, Public Health Evidence South Asia (PHESA), Manipal University, Manipal;

*theo.lakiang@gmail.com ;

**myrongodinho@gmail.com;

*** itzshruti@gmail.com;

[4]Assistant Professor, Public Health Evidence South Asia (PHESA), Manipal University, Manipal; meetbhumika123@gmail.com

Correspondence to:
Dr. N. Sreekumaran Nair
Director, Public Health Evidence South Asia (PHESA), Ground Floor, Manipal EDU Building, and Professor of Biostatistics and Head, Department of Statistics,
Level 6, Health Science Library Building,
Manipal University, Manipal-576104, Karnataka, India
Email address: sree.nair@manipal.edu,nsknairmanipal@gmail.com
Telephone number: +91-8202922407

For peer review only

Risk factors and barriers to case management of neonatal pneumonia: Protocol for a pan-India qualitative study of stakeholder perceptions

Abstract

Introduction: India accounted for more neonatal deaths (est. 696,000) than any other country, as of 2015. Of these deaths, most neonatal deaths due to infections can be attributed to pneumonia which accounts for 16% of all neonatal mortalities (2010). Despite simple, inexpensive case management strategies being available, pneumonia continues to cause significant mortality and morbidity among neonates. Understanding the perceptions and experiences of stakeholders of neonatal care can help find solutions to barriers to care and design tailored strategies for controlling neonatal pneumonia.

Methods and analysis: A pan-India qualitative study will be conducted. Participants include health care providers, programme officers, academicians, representatives of NGOs/ bilateral agencies and policy makers. They will be recruited purposively from rural and urban, public and private, and facility and community health care settings across six Indian regions. Within each region, a minimum of one state will be selected. Districts will be based on neonatal mortality indicators, and will be subject to feasibility at the time of conducting the study. We plan to conduct in-depth interviews (IDI) and Focus Group Discussions (FGD) focusing on (a) burden, (b) risk factors, (c) case management, (d) treatment guidelines, (e) barriers to case management, (f) recommendations. The number of interviews will depend on the information saturation. Interviews will be audio- recorded with prior written consent and transcribed verbatim. Principles of thematic analysis will be applied for qualitative data analysis on RQDA.

Ethics and dissemination: The protocol has been approved by the Health Ministry Screening Committee (HMSC), Government of India and the Institutional Ethics Committee at the host institution. Confidentiality and privacy of the participants will be maintained. The findings of the study will be shared with all stakeholders of this research including the participants. Knowledge dissemination workshops will be conducted to ultimately transfer the evidence tailored to the stakeholders' need.

Keywords: Neonate, Pneumonia, Qualitative study, Protocol, Case Management, India

For peer review only

Strengths and limitations of the study

- A pan-India study covering all regions of India and wide range of stakeholders of the public and private, rural and urban neonatal health system
- Contribution of knowledge about barriers to management and control of pneumonia among neonates in the Indian context
- Final selection of states and districts from each region will depend on the feasibility of access and willingness of participants in the district.

For peer review only

Introduction

In recent decades in India, the neonatal mortality rate has declined more slowly than the post-neonatal mortality rate, and now over half of India's under-five deaths occur in the neonatal age group. Seventeen percent of these are attributed to pneumonia, making it the most deadly neonatal infection.[1] In the international effort to curb child deaths, addressing neonatal pneumonia in India is clearly an issue of global concern.

Developing countries contribute to around 151 of the annual 156 million under-five pneumonia cases. India is the largest contributor to global pneumonia deaths, accounting for around 43 million cases per year. Neonatal pneumonia differs from childhood pneumonia in many ways, and is classified as 'early onset' or 'late onset', corresponding to the two major environments from which the infectious agent may be contracted: the intrauterine environment and birth canal, and the external environment. [2] Moreover, differences in infective agents result in a number of characteristic variants of neonatal pneumonia.[3]

The likelihood of occurrence can be augmented by risk factors and determinants. In India, economic compromise, poor immunization status, malnutrition and indoor air pollution are some of the major risk factors for developing pneumonia.[4] Studies have previously reported that low birth weight, inadequate antenatal care, home birthing, untrained personnel, and other factors are associated with neonatal pneumonia.[5]

Effective case management is a cornerstone of pneumonia control in children. However, studies have reported barriers (e.g. cost and complexities in treatment) to its implementation, especially in low-and-middle income (LMICs) like India, where the burden of pneumonia morbidity and mortality is high.[6] This is compounded by numerous supply (e.g. health system, staff absenteeism, lack of infrastructure) and demand (e.g. local social fact cultural barriers, health care perceptions and experiences) issues, which plague public health systems in developing countries.[7 8]

Understanding context-specific factors and barriers for management and control of neonatal pneumonia is essential when designing effective strategies. Each stakeholder at different levels of healthcare system (policymakers, healthcare providers, grass root level workers, informal care givers) experiences a different functional position in the healthcare

delivery system. Therefore, they are able to offer in-depth, diverse and comprehensive perspectives of neonatal care, all of which are essential to attain a more complete understanding of neonatal pneumonia. The holistic understanding may help address neonatal health by involving multiple actors and, thus, multiple factors to address neonatal health, a 'complex' problem. [9] Qualitative research has successfully been used to provide evidence to inform government-stated policy priorities in developing countries, by promoting an understanding of local problems and solutions to develop effective and sustainable interventions.[8]

Qualitative studies have previously focused on perceptions of caregivers on barriers and factors related to pneumonia in childhood. However, empirical evidence largely exists for the childhood pneumonia and neonatal sepsis. There is a need for a study which captures the variety and depth of context-specific perceptions and experiences associated with neonatal pneumonia in India's various regions.

Aims: This qualitative study is a part of a larger mixed-methods research project which also involves three systematic reviews on the same domains of inquiry as this study. This study will provide an in-depth and comprehensive understanding of neonatal pneumonia from stakeholders at different health system levels and settings across India. The findings of this study are planned to be synthesized with the evidence from the three reviews. The synthesized findings are anticipated to fill the evidence gap related to an understanding of the occurrence and case management of neonatal pneumonia in the Indian context. In addition, the recommendations provided by the stakeholders from their experience will serve as essential inputs to inform policy and design strategies to address neonatal health disparities more comprehensively across the diverse range of contexts present in this country.

Objectives:

1. To identify the risk factors of pneumonia in the neonatal age group (0-28 days) in the Indian context.

For peer review only

2. To understand pertinent barriers to the case management of neonatal pneumonia in Indian health care facilities.
3. To understand factors that could facilitate the control of neonatal pneumonia in India

Methods and analysis:

Research team and Reflexivity:

Three adequately qualified and trained researchers (Female: SM; Male: MG, TL) will conduct the in-depth interviews and/or focus group discussions. SM is qualified in dentistry (BDS) and public health (MPH), MG is a medical doctor (MBBS) with public health training and TL is qualified in nursing (BSN) and public health (MPH). SM and TL had prior experience in qualitative research. All three researchers were working research associates for this project, and received training qualitative research from methodological experts (Faculty of Spatial Sciences, University of Groningen, The Netherlands; PRAYAS, Pune, India), especially for in depth interviews and focus group discussions.

Regarding the language proficiencies of the interviewers, all three have native-level proficiency of English (read/write/speak) for stakeholders who would prefer English as the medium of conversation. Two of the researchers have native-level proficiency of Hindi (read/write/speak), the second official language (after English) of India, for stakeholders who would prefer Hindi as the medium of conversation. One researcher has native-level proficiency (read/write/speak) of languages of West (e.g Marathi), South (e.g. Kannada, Tamil), and one researcher has native-level proficiency of languages and dialects of North-East India. Considering that this is a pan-India study, we acknowledge that there could arise a situation where language may become a barrier and introduce a bias. Thus in specific states where we do not possess sufficient linguistic skills for an interview, e.g. Telangana and Andhra Pradesh, Gujarat/ Rajasthan, we have established networks and contacts who would provide us with trained interpreters on request for facilitating the interview. We will report such instances in the final manuscript after results in the section on "Reflexivity" for information of the readers.

For peer review only

Study Design: A qualitative study will be conducted to explore and understand the perceptions and experiences of various stakeholders regarding the following domains of interest:

- risk factors associated with neonatal pneumonia in the Indian context;
- barriers to the case management of neonatal pneumonia;
- recommendations for control and management of neonatal pneumonia in India

Study Setting: India, the largest country in South Asia, is the world's second most populous country, with about 1.03 billion people in 29 States and 7 Union Territories.[10 11] Eleven percent of the total population is under 4 years of age.[1] Over two-thirds of the population live in rural areas which continue to lag far behind urban India in socioeconomic progress.[12] This disparity is reflected in the neonatal health statistics: while Uttar Pradesh has a neonatal mortality rate (NMR) of 49 deaths per thousand live births (DPTLB) which is higher than the regional aggregate for sub-Saharan Africa, Kerala boasts a single digit NMR of 7 DPTLB, resembling a considerably more developed nation.[13] Even within a state, statistics vary greatly between districts;[14] with Shrawasti and Hamirpur illustrating the contrast in Uttar Pradesh, with NMRs of 50.8 and 17.6 DPTLB, respectively.[15]

Participants

Through discussions with advisory members, it was agreed that as far as possible, we would target stakeholders from every component and level of the health system. We aim to include the following stakeholders in our study:

1. Programme officers (e.g. RMNCH+A/ IMNCI, State/District Health Officers)
2. Neonatologists, Pediatricians, Medical officers, Neonatal nurses, GNMs (General Nurse Midwives), ANMs (Auxiliary nurse midwives) and other facility-based staff.
   It is anticipated that much information can be obtained on risk factors for neonatal pneumonia (hospital-acquired and ventilator-associated) and barriers to facility-based case management of neonatal pneumonia.
3. Community health workers (e.g. Accredited Social Health Activists [ASHA] and state-specific equivalents), AnganWadi Workers (AWW).
   It is anticipated that community-based staff will provide valuable insights into the burden, risk factors and barriers to the case management of neonatal pneumonia, as well as other related health services and schemes available for neonatal care.

For peer review only

4. NGO/ bilateral agencies (e.g. UNICEF) representatives.
5. Academicians (e.g. Indian Academy of Pediatrics' members)
6. Policymakers (e.g. Ministry of Health and Family Welfare representatives)

Sampling strategy

This study will be conducted during 2016-17 (details on specific data collection periods have been provided in the section on "study status" below). We will recruit participants from multiple regions within India, representing both urban and rural, as well as facility-based and community-based neonatal care. To achieve this, we adapted a classification proposed in a previous study on neonatal mortality in India, where states were grouped into six regions: North, South, Central, West, East and Northeast.[16]. Within each region, a minimum of one state will be selected. Using the most recent neonatal mortality estimates available,[15] we generated a series of district-level maps of neonatal mortality indicators (published elsewhere). Within the selected state, districts were grouped by NMR from published secondary data. We identified a total of four districts in each state: two with the lowest NMR (best performers), and two with the highest NMR (worst performers) (Figure 1). We plan to recruit participants from one of each of the best performing and worst performing districts respectively. All geographical areas suspected to be conflict-prone, unsafe or inaccessible will be identified and excluded. District selection will also be subject to feasibility (e.g. after contacting key informants and liaisons in identified districts and discussing with advisory committee members). From these district(s), participants from urban and rural, public and private facility and community health care settings at different levels of the health care system will be recruited. This strategy, despite being time- and labour-intensive, was developed to recruit participants from contrasting settings, allowing for a wider range of perspectives, in order to build a comprehensive picture of neonatal pneumonia in India.

Recruitment: A purposive sampling approach will be employed to include participants from different settings and at various levels of the health system. Health care providers from private and public setups, at the community and facility at the primary, secondary and tertiary health care level will be included. Participants at different health system levels i.e. responsible for policy making and implementation will also be identified and recruited.

For peer review only

To ensure successful participation, participants will be identified by liaising with governmental, non-governmental, bilateral and academic organizations who work in maternal and child health fields in the selected regions. Contact networks of the project and advisory members will also be utilized to facilitate this process. The participants will then be invited through personal telephonic calls and a formal invitation letter which will include the project brief. The participants will be informed about the project goals and provided with a project brief via email. Additionally, participants will also be informed about the interviewers' background and the reasons for conducting this research.

On consenting to participate in the research, the time, location and mode (face-to-face/ tele-interviews) of IDI will be ascertained through consultation with the participants. All efforts will be made to interview the participant in their natural setting. We plan to include a minimum of 24 neonatologists/ pediatricians, 12 neonatal or staff nurses, 12 community health workers (ANM/ASHA), 12 medical officers and 6 policy makers will be interviewed in total. However, the final numbers will be determined by attaining information saturation.

Data collection methods: In-depth interviews (IDI) and focus group discussions (FGD) will be used to collect data. While IDI will comprise the main mode of data collection, we anticipate that focus group discussions (FGDs) might become necessary in some instances where nurses or community health workers may be involved as participants. When a minimum of four participants of the same category (e.g. nurses or ASHAs) are available and in depth interviews may not seem feasible (e.g. time constraints per participant), a FGD will be conducted. Similar guides will be used for IDI and FGD.

A detailed IDI/FGD guide will be formulated after literature review, in consultation with clinical experts and qualitative research specialists. The guide will be customized to the level of the stakeholder to allow questioning strategy to be more flexible. The IDI/FGD guide which will contain the lists of issues we want to explore and will include open ended questions on the following themes relating to neonatal pneumonia in India (a) burden, (b) risk factors, (c) case management, (d) treatment guidelines, (e) barriers to case management, (f) recommendations. The guide for the community health workers will focus on community-acquired pneumonia in neonates and additionally include other relevant health schemes/ services related to community-acquired neonatal pneumonia in India.

For peer review only

Supporting documents will include development of (i) survey form which collects data on participant details (socio-demographic information and duration of experience), consent details and interview details, and (ii) observations and field notes summary form. The guide will be pilot-tested before we proceed with the data collection in order to ensure that the guide is vivid and participants understanding of the concept and language. Any modification required will be taken into consideration after pilot-testing of the guide to be able to generate more in-depth information. Interviews will be conducted either through face to face interaction or through online sources like Skype, teleconference, etc. (based on the convenience of the interviewee). During the interview, the primary researcher will lead the interview, and a second researcher will be responsible for recording the interview, making observations and taking field notes.

Recording: Interviews will be conducted in a language which the participant is comfortable speaking. Each interview is anticipated to last for 60 to 90 minutes. The number of interviews per participant will depend on information saturation. For face-to-face interviews, an interview and a note taker will be present at each interview. For tele-interviews, only the interviewer will be present at each interview. Interviews will be audio-recorded on a portable, electronic voice recorder (Sony ICD-PX440), and transferred to a computer for storage and transcription. Field notes will be maintained for every interview which will highlight the reflections on the interview process, non-verbal cues of the interviewee as well as the initial themes that emerge as a result of the interview.

Data management

Data organization: A unique identification code will be assigned to audio files. Audio recording of each interview will be assigned with a similar unique identification code to the participant's identification code. Naming of the file will help the team to easily locate and retrieve the file whenever required for transcription. All transcripts will be named after participation ID and date and place of interview. Then the transcripts of each interview will be saved along with the informed consent of that particular participant.

Data transcription: All interviews will be recorded (with prior consent) and transcribed verbatim (involves active and repeated listening and observation of the data collected). Interviews will first be transcribed in the language of the interview and then translated into

For peer review only

English. The transcripts will be supplemented with field notes, if and when necessary. The English transcripts for interviews other than those in English will be back translated into that language at a later stage for quality assurance purposes. To ensure quality, 10% of the transcripts will be checked with the tape-recorded interviews by a researcher other than the interviewer.

Data storage: A master archival log and an archival information sheet will be developed beforehand, all audio files and transcripts lists will be entered into the archival sheet. The archival sheet such will contain details such as date, location, interviewer's name, name of the person who transcribed the data etc. Backup of all transcript files to a secured external device which can be accessible only to the research team

Data protection: Audio files, transcripts, etc. will be shared only amongst the research team and as required by regulatory laws of the Government of India. The files will be password protected and can only be accessible by agreed members of the research team. Computer files need to be kept securely and can only be shared based on the terms and condition that the participant agreed upon in the informed consent.

Data ownership: Research team, funding agency and stakeholders will retain ownership of the data. Further clarification on data ownership will be discussed.

Data Analysis: We will follow a grounded theory approach. The transcript will be cleaned only when it has to do with the identity of the participants. Where possible, the data from the transcripts will be organized in the form of a chart or table that will data allows us to easily analyze the response to each question individually, in order to make it easier to pick out concepts and themes. Thematic analysis of the transcripts will be done. To address the viewpoints and opinions collected from different stakeholders (e.g. pediatricians, community health workers, policy makers), we will also analyze the responses according to the type of stakeholder.

Codes will be developed by reading and re-reading the transcripts. For this, specific words/phrases/ideas/opinion/issues related to the objective of the study that occurs repeatedly in the data will be identified. The interviewers will make note of the different ideas as the different responses are read through. Different codes will be explored and

For peer review only

listed till information is saturated. A code book will be developed to list and define the codes identified based on the objective of the study. Two of the research team members will code the data, and an independent qualitative research advisor of the project will check the two cycles of coding using the IDI guide as reference. The coders will then meet to discuss the coding schemes and resolve differences. Re-labeling of the codes will be done if and where necessary. Next, similar codes will be grouped and categorized in a meaningful way. This will subsequently lead to generation of important categories and concepts related to the main themes of the study. Each response may be associated to one or more theme. Hence, different categories can be classified under one main over-arching theme. In addition to already identified themes, themes will be derived, if any, from the categories. The data will be managed by the use of R QDA package. This software will facilitate the organization and management of datasets at every stage of qualitative data analysis.

Ethics and dissemination

Ethical Approval: The study will be conducted according to the principles in the Declaration of Helsinki. The protocol has been approved by the Health Ministry Screening Committee (HMSC), Ministry of Health and Family Welfare (MoHFW), Government of India and the Institutional Ethics Committee at Manipal University, Manipal, India (i.e the host institution).

Informed Consent: Participation will be entirely voluntary. Informed consent will be mandatory for participation in the study. For face-to-face interviews, written informed consent will be obtained. For tele-interviews, verbal informed consent will be obtained. Where recording is denied, hand written notes will be taken by the researchers. [17]

Confidentiality: Confidentiality and privacy of the participant will be maintained at every stage of the research. A unique identification code will be given to each participant. Recordings of the interviews will be assigned only the number and cannot be linked to the participant's identifiers. During data cleaning, all potential identifiers will be removed to create a 'clean' data set.[18] The data will be kept anonymous at all stages of the study. Participants will remain unidentifiable in all transcripts, and in all reports from this study.

Dissemination: The findings of the study will be shared with all stakeholders of this research including the participants. Knowledge dissemination workshops will be conducted with

For peer review only - http://bmjopen.bmj.com/site/about/guidelines.xhtml

relevant stakeholders to ultimately transfer the evidence tailored to the stakeholder (e.g. policy briefs, publications, information booklets, etc).

Strengths and limitations of the study

Strengths: This is a pan-India study covering all regions of India and wide range of stakeholders of the public and private, rural and urban neonatal health system. This is the first qualitative study, to the best of our knowledge, to address the issue of neonatal pneumonia in India. Thus, this study will contribute to the knowledge about prevention, management and control of pneumonia among neonates in the Indian context.

Limitations: There are some limitations to our study. Final selection of states and districts from each region will depend on the feasibility of access and willingness of participants in the district. Secondly, we have not included parents/ guardians as potential participants. Third, we anticipate that there may exist a perceived power difference (e.g. in qualification, professional status, etc) between the interviewer and interviewee (e.g. policy makers, programme managers, pediatricians), which may limit the depth of data collected. We have tried to counter this limitation with extensive training and mock interviews with similar stakeholders. Additionally, as required by COREQ, we will report each interviewee's characteristics.

Quality Control and Reporting

Reporting of the qualitative study will be informed by two guidelines: The Standards for Reporting Qualitative Research (SRQR)[19] and the Consolidated criteria for reporting qualitative research (COREQ).[20]

Study status

The data collection has not yet been completed for this study. The data collection has been planned in two phases to cover 6 regions of India. The first phase covering 4 regions was done from April- June 2016. Recruitment was done in the month of April-May 2016. Data collection was performed from 17 May 2016 to 5 June 2016.

The second phase is planned during July-September 2017. Recruitment and contact with stakeholders is ongoing (June 2017) for finalization of specific dates. Data collection has been planned during third week of August 2017 and mid-September 2017 for the remaining regions.

Acknowledgements: We would like to thank the following individuals for their continuous support and guidance during this process of protocol development: Dr. Ajay Bailey, Assistant Professor, Faculty of Spatial Sciences, University of Groningen, The Netherlands; Dr.

For peer review only

Shrinivas Darak, Senior researcher, PRAYAS, Pune, Maharashtra; Dr. Manoj Das, Director Projects, The INCLEN Trust International, New Delhi; Dr. B. Shantharam Baliga, Professor, Department of Paediatrics, Kasturba medical college, Mangalore, Karnataka; Dr. K.K. Diwakar, Professor and Head, Department of Neonatology, Associate Dean, Malankara Orthodox Syrian Church Medical College, Kerala and Dr. Unnikrishnan B., Associate Dean and Professor, Department of Community Medicine, Kasturba Medical College, Mangalore. We also thank Public Health Evidence South Asia (PHESA) and Manipal University, Manipal for providing the necessary institutional and infrastructural support for the project. We would like to thank The INCLEN Trust International, New Delhi, and The Bill and Melinda Gates Foundation for the financial support which made this project possible.

Contributorship statement: SN, BV and LL conceived the research idea and reviewed the manuscript. SN and LL provided overall technical guidance. In addition, LL assisted in developing the in-depth interview (IDI) guides. TL, MG and SM designed the protocol, drafted the manuscript, and developed and pilot-tested the IDI guides. All authors approved the final version of the manuscript.

Funding: This project is supported by a grant from Bill and Melinda Gates Foundation (grant OPP1084307) to The INCLEN Trust International and sub-grant to Manipal University (subgrant INC2015GNT004). However, the views expressed through this project do not necessarily represent the views of the Bill and Melinda Gates Foundation, The INCLEN Trust International, or Manipal University.

Competing Interests: All authors have completed the ICMJE uniform disclosure form at www.icmje.org/coi_disclosure.pdf and declare: all authors had financial support (grants) from Bill and Melinda Gates Foundation (grant OPP1084307) to The INCLEN Trust International and sub-grant to Manipal University (subgrant INC2015GNT004)., during the conduct of the study and for the submitted work; no financial relationships with any organisations that might have an interest in the submitted work in the previous three years; no other relationships or activities that could appear to have influenced the submitted work.

Data sharing statement: Due to ethical concerns, supporting data (e.g. interview transcripts) cannot be made publicly available. All other data supporting this study (e.g.

For peer review only

coding structure) will be provided as final results and as supplementary material accompanying the manuscript of the study’s final results

For peer review only

References

1. World Health Organisation (WHO). Neonatal and Child Health Profile, India: Department of Maternal Newborn Child and Adolescent Health 2013 [accessed 2016 19/1/2016]. Available from: http://www.who.int/

2. Dennehy P. Respiratory infections in the newborn. *Clinics in perinatology* 1987;14(3):667-82.

3. Webber S, Wilkinson A, Lindsell D, et al. Neonatal pneumonia. *Archives of disease in childhood* 1990;65(2):207-11.

4. Mathew JL, Patwari AK, Gupta P, et al. Acute respiratory infection and pneumonia in India: a systematic review of literature for advocacy and action: UNICEF-PHFI series on newborn and child health, India. *Indian pediatrics* 2011;48(3):191-218.

5. Choudhury A, Nargis S, Mollah A, et al. Determination of risk factors of neonatal pneumonia. *Mymensingh medical journal: MMJ* 2010;19(3):323-29.

6. UNICEF, World Health Organization. Global action plan for prevention and control of pneumonia (GAPP). 2009

7. Patil AV, Somasundaram K, Goyal R. Current health scenario in rural India. *Australian Journal of Rural Health* 2002;10(2):129-35.

8. Bedford KJA, Sharkey AB. Local barriers and solutions to improve care-seeking for childhood pneumonia, diarrhoea and malaria in Kenya, Nigeria and Niger: a qualitative study. *PloS one* 2014;9(6):e100038.

9. Rwashana AS, Nakubulwa S, Nakakeeto-Kijjambu M, et al. Advancing the application of systems thinking in health: understanding the dynamics of neonatal mortality in Uganda. *Health Research Policy and Systems* 2014;12(1):1.

10. The World Bank. World Bank Open Data. Rural Population. 2016 [accessed 2016 15/4/2016]. Available from: http://data.worldbank.org/.

11. Office of the Registrar General & Census Commissioner MoHA, Government of India. . Age structure and Marital Status, Census 2011. Available from: http://censusindia.gov.in/.

12. Mukunthan A. Rural India is far behind Urban India in every Indicator of Progress. Factly.[newspaper article on the Internet]. 2015. [accessed 2016 16/4/2016]. Available from: https://factly.in/rural-india-behind-urban-india-in-progress-indicators/

13. Ministry of Health and Family Welfare, Department of Health and Family Welfare, Governmnet of India.Annual Health Survey. Catalog of Indicators [accessed 2016 1/1/2016]. Available from: https://data.gov.in/catalog/indicators-annual-health-survey.

14. Ministry of Health and Family Welfare, Government of India (GoI). Child Health Division. India Newborn Action Plan. New Delhi; September 2014 [accessed 2016 14/1/2016]. Available from: http://www.newbornwhocc.org/.

15. Ram U, Jha P, Ram F, et al. Neonatal, 1–59 month, and under-5 mortality in 597 Indian districts, 2001 to 2012: estimates from national demographic and mortality surveys. *The Lancet Global Health* 2013;1(4):e219-e26.

16. Million Death Study Collaborators. Causes of neonatal and child mortality in India: a nationally representative mortality survey. *The Lancet* 2010;376(9755):1853-60.

17. World Health Organisation (WHO). Informed Consent Form Templates 2016 [accessed 2016 8/4/2016]. Available from: http://www.who.int/.

18. Kaiser K. Protecting respondent confidentiality in qualitative research. *Qualitative Health Research* 2009;19(11):1632-41.

19. O'Brien BC, Harris IB, Beckman TJ, et al. Standards for reporting qualitative research: a synthesis of recommendations. *Academic Medicine* 2014;89(9):1245-51.

20. Tong A, Sainsbury P, Craig J. Consolidated criteria for reporting qualitative research (COREQ): a 32-item checklist for interviews and focus groups. *International journal for quality in health care* 2007;19(6):349-57.

For peer review only

Figure Caption/ legend

Figure 1. A map of India depicting the two best and worst performing districts, by NMR, in each Indian state.

Source: adapted, with permission, from data published by Ram U, Jha P, Ram F, et al. Neonatal, 1–59 month, and under-5 mortality in 597 Indian districts, 2001 to 2012: estimates from national demographic and mortality surveys. The Lancet Global Health 2013;1(4):e219-e26.

For peer review only

State/ UT	NMR	WPD	NMR	BPD	NMR
**Northern Region**					
**State/ UT**	**NMR**	**WPD**	**NMR**	**BPD**	**NMR**
Punjab	23.4	Barnala	36.8	Shahid BSNagar	10.2
		Muktsar	34.6	Fatehgarh Sahib	9
Himachal Pradesh	27.5	Kangra	38.1	Hamirpur	17.9
		Sirmaur	36.7	Mandi	15.6
Haryana	26.9	Mewat	33.5	Mahendragarh	19.6
		Sirsa	32.1	Sonipat	19.5
Uttarakhand	24.9	Champawat	34.7	Almora	15.4
		Hardwar	31.4	Rudraprayag	15.2
Jammu & Kashmir	30.1	Kargil	58.1	Udhampur	15.6
		Kupwara	55.6	Jammu	14.4
Chandigarh	15.5	*(Union Territory)*			
**Central Region**					
**State/ UT**	**NMR**	**WPD**	**NMR**	**BPD**	**NMR**
New Delhi	19	North-west Delhi	58.3	Central Delhi	10
		New Delhi	24.9	South Delhi	17.6
Madhya Pradesh	39.8	Datia	65.1	Sehore	28
		Panna	53.9	Indore	19
Uttar Pradesh	39.3	Shrawasti	50.8	Deora, Saharanpur	27.8
		Lalitpur	50.2	Azamgarh	27.2
Rajasthan	36.5	Tonk	47.9	Alwar	28.6
		Bundi	47.7	Dungarpur	27.2
Chhattisgarh	36.1	Korba	48.2	Durg	27.9
		Bastar	46.3	Raipur	27.2
**Western Region**					
**State/ UT**	**NMR**	**WPD**	**NMR**	**BPD**	**NMR**
Maharashtra	21.6	Chandrapur	37.9	Ratnagiri	12.6
		Gondiya	35	Mumbai	12
Goa	7.9	North Goa	9.7	South Goa	6.3
Gujarat	29	Patan	43.2	Surat	17.6
		Dohad	42.1	Narmada, Tapi	17.2
**Eastern Region**					
**State/ UT**	**NMR**	**WPD**	**NMR**	**BPD**	**NMR**
West Bengal	24.2	Koch Bihar	34.3	Hugli	16.6
		Uttar Dinajpur	33.3	Purba Medinipur	11.1
Bihar	29.5	Arwal	36.9	Sheikhpur	22.1
		Jehanabad	35.2	Siwan	17.6
Jharkhand	27.7	Khunti	41.9	Giridh	22.6
		Pakaur	41.2	Jamatara	17.2
Orissa	38.4	Kandhamal	56.2	Bhadrak	26.3
		Rayagada	55.5	Puri	25
**North-east Region**					
**State/ UT**	**NMR**	**WPD**	**NMR**	**BPD**	**NMR**
Arunachal Pradesh	30.6	Papum Pare	45.7	Anjaw	16.4
		Lower Subansiri	42.1	Lower Dibang Valley	16
Assam	31.9	Udalgiri	48.2	Tinsukia	15.9
		Karumganj	48	Dima Hasao	10.9
Tripura	29.8	*(Smaller States)*			
Meghalaya	21.3				
Nagaland	17.8				
Sikkim	17.4				
Manipur	16.9				
Mizoram	14.7				
**South Region**					
**State/ UT**	**NMR**	**WPD**	**NMR**	**BPD**	**NMR**
Andhra Pradesh	29.6	Kurnool	40.5	Warangal	18
		Adilabad	39.6	Hyderabad	17.7
Karnataka	23.2	Raichur	39.3	Bangalore	11.8
		Koppal	38.1	Udupi	8
Tamil Nadu	15.3	Theni	23.8	Erode	10.9
		Madurai	23.4	Chennai	9.1
Kerala	7.3	Wayanad	13.4	Ernakulam	5.1
		Alappuzha	11.5	Pathanamthitta	5.1
Puducherry	14.9	*(Union Territory)*			

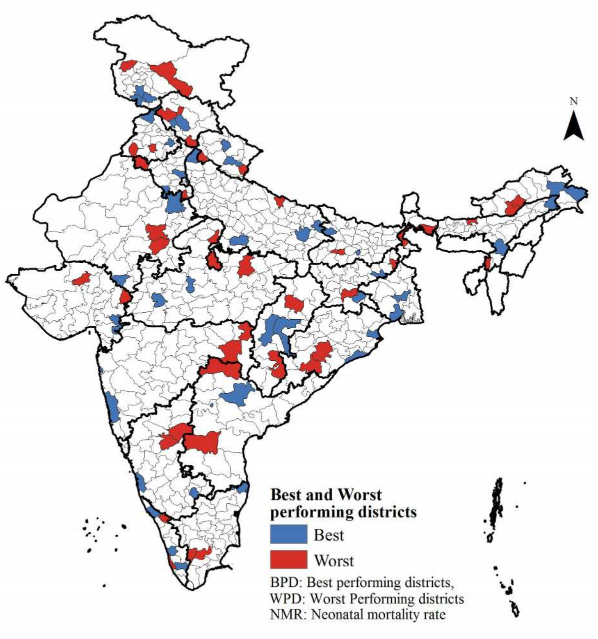

Figure 1. A map of India depicting the two best and worst performing districts, by NMR, in each Indian state. Source: adapted, with permission, from data published by Ram U, Jha P, Ram F, et al. Neonatal, 1–59 month, and under-5 mortality in 597 Indian districts, 2001 to 2012: estimates from national demographic and mortality surveys. The Lancet Global Health 2013;1(4):e219-e26.

420x297mm (300 x 300 DPI)

Compliance with the "Consolidated criteria for reporting qualitative studies (COREQ): 32-item checklist"

Sl. No	Item	Guide questions/ description	Compliance (pg)
**Domain 1: Research team and reflexivity**			
**Personal Characteristics**			
1.	Interviewer/facilitator	Which author/s conducted the interview or focus group?	6
2.	Credentials	What were the researcher's credentials? *E.g. PhD, MD*	6
3.	Occupation	What was their occupation at the time of the study?	6
4.	Gender	Was the researcher male or female?	6
5.	Experience and training	What experience or training did the researcher have?	6
**Relationship with participants**			
6.	Relationship established	Was a relationship established prior to study commencement?	8,10
7.	Participant knowledge of the interviewer	What did the participants know about the researcher? *e.g. personal goals, reasons for doing the research*	9
8.	Interviewer characteristics	What characteristics were reported about the interviewer/facilitator? *e.g. Bias, assumptions, reasons and interests in the research topic*	NA
**Domain 2: study design**			
**Theoretical framework**			
9.	Methodological orientation and Theory	What methodological orientation was stated to underpin the study? *e.g. grounded theory, discourse analysis, ethnography, phenomenology, content analysis*	11
**Participant selection**			
10.	Sampling	How were participants selected? *e.g. purposive, convenience, consecutive, snowball*	7-10
11.	Method of approach	How were participants approached? *e.g. face-to-face, telephone, mail, email*	9
12.	Sample size	How many participants were in the study?	NA
13.	Non-participation	How many people refused to participate or dropped out? Reasons?	NA
**Setting**			
14.	Setting of data collection	Where was the data collected? *e.g. home, clinic, workplace*	10, 11
15.	Presence of non-participants	Was anyone else present besides the participants and researchers?	NA
16.	Description of sample	What are the important characteristics of the sample? *e.g. demographic data, date*	NA
**Data collection**			
17.	Interview guide	Were questions, prompts, guides provided by the authors? Was it pilot tested?	9, 10
18.	Repeat interviews	Were repeat inter views carried out? If yes, how many?	NA
19.	Audio/visual recording	Did the research use audio or visual recording to collect the data?	10
20.	Field notes	Were field notes made during and/or after the interview or focus group?	10,11
21.	Duration	What was the duration of the inter views or focus group?	10
22.	Data saturation	Was data saturation discussed?	9
23.	Transcripts returned	Were transcripts returned to participants for comment and/or correction?	NA
**Domain 3: analysis and findings**			
**Data analysis**			
24.	Number of data coders	How many data coders coded the data?	12

For peer review only

25.	Description of the coding tree	Did authors provide a description of the coding tree?	NA
26.	Derivation of themes	Were themes identified in advance or derived from the data?	NA
27.	Software	What software, if applicable, was used to manage the data?	12
28.	Participant checking	Did participants provide feedback on the findings?	NA
**Reporting**			
29.	Quotations presented	Were participant quotations presented to illustrate the themes/findings? Was each quotation identified? *e.g. participant number*	NA
30.	Data and findings consistent	Was there consistency between the data presented and the findings?	NA
31.	Clarity of major themes	Were major themes clearly presented in the findings?	NA
32.	Clarity of minor themes	Is there a description of diverse cases or discussion of minor themes?	NA

Source: Tong A, Sainsbury P, Craig J. Consolidated criteria for reporting qualitative research (COREQ): a 32-item checklist for interviews and focus groups. International Journal for Quality in Health Care. 2007. Volume 19, Number 6: pp. 349 – 357

For peer review only
